# Investigation of Self-Healing Performance of Asphalt Mastic—From the Perspective of Secondary Aging

**DOI:** 10.3390/ma16247567

**Published:** 2023-12-08

**Authors:** Bo Li, Yu Wang, Peng Xiao, Aihong Kang, Yao Zhang, Zhengguang Wu

**Affiliations:** 1 College of Civil Science and Engineering, Yangzhou University, Yangzhou 225127, China; libo@yzu.edu.cn (B.L.); yuwang4012@126.com (Y.W.); ahkang@yzu.edu.cn (A.K.); yaozhang@yzu.edu.cn (Y.Z.); zgwu@yzu.edu.cn (Z.W.); 2 Research Center for Basalt Fiber Composite Construction Materials, Yangzhou 225127, China

**Keywords:** asphalt mastic, recycling, fatigue self-healing, fracture self-healing, secondary aging

## Abstract

Reclaimed asphalt pavement (RAP) has been widely utilized because it is an environmentally friendly and economical material. The performance of recycled asphalt mixtures will deteriorate gradually with the secondary aging process of asphalt, including the self-healing property. To further understand the self-healing characteristics of asphalt after secondary aging, taking 70# petroleum asphalt, SBS-modified asphalt, and extracted old asphalt mastics as objects, the fatigue self-healing test and fracture self-healing test were conducted to simulate the intermediate-and low-temperature healing behaviors of different asphalt mastics. The impact of healing time, healing temperature, and aging degree of mastics on the healing performance was systematically investigated. The results show that the original unaged asphalt mastics present excellent fatigue healing properties with an index of 0.796 and 0.888 for 70# petroleum and SBS-modified asphalt mastics, respectively. The secondary aging process causes significant impact on the healing properties, leading to a great drop in the corresponding index, which decreased to 47.5% and 72.5% of that of the unaged ones. The fracture healing ability of all mastics was much inferior to the fatigue healing. After secondary aging, the fracture healing index values of 70# petroleum asphalt, SBS-modified asphalt, and extracted old asphalt mastics were all as low as around 0.3, indicating similar performance can be found in the secondary aged SBS-modified asphalt mastics and 70# asphalt mastics. Overall, after secondary aging, the fatigue damage of SBS-modified asphalt mastics can be cured effectively by self-healing, but the fatigue and fracture self-healing properties of 70# asphalt mastics are difficult to recover. These results could provide an innovative view to understand the fatigue and fracture healing characteristics of recycled asphalt pavement after secondary aging.

## 1. Introduction

Reclaimed asphalt pavement (RAP) has been gradually widely used because of its advantages of saving resources and reducing pollution. However, recycled asphalt mixtures containing a high amount of RAP normally present deteriorated performance, especially in the fatigue cracking and low temperature cracking resistance [1,2].

The self-healing performance of recycled asphalt is an important aspect to address due to its comprehensive performance. Self-healing refers to the ability of a material to repair itself when damaged by the environment or external force. As an elastic–plastic material, asphalt has a certain self-healing characteristic. During the rest period without vehicle load, asphalt will present obvious self-healing behavior and repair the micro cracks inside the pavement, so as to restore the pavement performance and prolong the service life [3]. Therefore, the study of the self-healing performance of recycled asphalt for the mixture design is of great significance. Since asphalt is a viscoelastic material with complex compositions, there is no unified theory for the study of asphalt self-healing mechanism. At present, the mainstream self-healing mechanisms include molecular diffusion energy, surface energy theory, capillary flow theory [4,5,6,7,8], etc.

Based on these theories, many evaluation methods and indicators have been derived. Through fatigue-healing-fatigue tests, three-point bending tests, and linear amplitude sweep test, cumulative dissipation energy and (or) tensile fracture stress-based healing indexes were proposed [9,10]. In addition, many scholars have also studied the influencing factors of asphalt self-healing performance, mainly including healing time, healing temperature, asphalt type, and damage degree, etc. Through the above experiments, a general conclusion is drawn that after aging, the lightweight components decrease and the asphaltene and resin contents increase, making the aged asphalt harder and leading to deterioration in flowability and self-healing performance.

(1) Healing time

Some scholars introduced healing time into the study of asphalt fatigue characteristics. Norambuena-Contreras et al. [11] found that there is an optimal healing time for the self-healing of asphalt. When the healing time is too short, the cracks cannot be completely repaired. When the healing time is too long, it leads to the aging of asphalt and reduces its mechanical properties. Zhang et al. [12] have found that the fatigue resistance of asphalt mixtures is significantly improved by introducing a certain healing time after loading. Therefore, the growth in fatigue life can be used to evaluate its healing performance.

(2) Healing temperature

The healing temperature is of great significance to the self-healing performance of asphalt. Sun et al. [13] used the molecular dynamics model to explain the effect of temperature on the self-healing ability of asphalt. The optimal healing temperature range of asphalt is determined by differential scanning calorimetry (DSC), which is 40.3–48.7 °C. Grossegger et al. [14] used different healing temperatures to study the capillary flow of asphalt. The results show that the surface energy and contact angle of asphalt decrease with the increase of temperature, resulting in faster capillary flow of asphalt. Combined with the additional pressure caused by the increase in temperature, the self-healing performance of asphalt is greatly enhanced. Fan et al. [15] evaluated the thermal sensitivity of asphalt mixtures by microwave heating and analyzed the self-healing performance before and after microwave heating by semi-circular bending fracture healing test, the experimental results indicate that the self-healing performance of asphalt mixtures is directly proportional to temperature, but the healing rate gradually slows down.

(3) Asphalt type

Scholars hold different views on the influence of modifiers on the self-healing performance of asphalt. Ding et al. [16] found through their research that the bonding performance of rubber modified asphalt is significantly improved, which can effectively prevent the occurrence of cracks and repair fine cracks better than unmodified asphalt. Xu et al. [17] studied the influence of different modifiers on the self-healing performance of asphalt. The results show that no matter what modifier is used, the increase of modifier content will reduce the self-healing ability of modified asphalt, which proves that petroleum asphalt possesses superior self-healing ability. Zhou et al. [18] selected five representative modified asphalts and petroleum asphalts to compare their self-healing properties. The experimental results show that all modifiers have a negative effect on the self-healing capability of asphalt in the early stage, but with the increase of healing time, self-healing ability becomes comparable for the modified and petroleum asphalt. Fakhri et al. [19] developed a nano-silica modified asphalt, which shows better self-healing performance than neat asphalt when the amount of modifier is less than 8%. Teknologi and Arshad et al. [20,21] experimentally investigated the performance of nano-modified asphalt, and the results indicate that nano modification can be one of the effective ways to improve the self-healing and road performance of asphalt.

(4) Damage Degree

Damage degree is another important impact factor on the self-healing performance of asphalt. Li et al. [22] used DSR fatigue test to simulate different damage degrees of asphalt. The results show that the healing index increases with the decrease of damage degree, which also can be simulated by Ramberg–Osgood model. Lv et al. [23] developed a new calculation formula based on the calculation of conventional self-healing index. Based on the rest-damage superposition principle (RDSP), in which the linear amplitude sweep frequency test (LAS) was used to quantify the self-healing performance of asphalt binders with different degrees of damage and rest periods, the ratio of loading times required to reach the same modulus before and after healing was calculated. Finally, a self-healing performance prediction model was established, with the fitting degree of over 0.9. Zhao et al. [24] explored the multiple damage healing laws of laboratory-simulated recycled asphalt by dynamic shear rheometer (DSR), fourier transform infrared (FTIR), thermogravimetric analysis (TGA), and gel permeation chromatograph (GPC). The results show that the cycles of damage-healing times had a greater impact on the healing performance than the healing time. Wang et al. [25] used different loading times to simulate different damage degree, and the test results show that if the asphalt is in minor damage state, the short-term self-healing performance will be better than the long-term performance. However, when the asphalt is in serious damage state (such as enduring low temperature fracture), the asphalt almost lost the healing performance.

(5) Aging degree

With the deepening aging of asphalt, the content of macromolecular compounds such as asphaltene and resin increases, which hinders the flow and diffusion between asphalt molecules and thus affects the self-healing performance of asphalt. Xiang et al. [26] investigated the internal structure of asphalt before and after aging by different microstructure tests. The final test data show that the self-healing performance of asphalt decreases with the increase in aging times, and the presence of rejuvenating agent can restore partly the self-healing performance of aged asphalt. Sun et al. [27] explored the chemical composition, colloidal stability, and macroscopic phase transition of asphalt aged in a laboratory. The results show that the self-healing sensitivity of asphalt to temperature decreases with the deterioration of aging degree.

Asphalt mastic is an essential component in asphalt mixture, of which the self-healing property directly affects the recovery ability of pavement performance. In particular, recycled asphalt pavement undergoes secondary aging during service, and the self-healing properties of asphalt mastic subsequently change significantly. However, there is a lack of understanding of the self-healing performance of recycled asphalt mastic after and before aging process.

In this paper, various types of asphalt mastics under different aging states were fabricated to study the changing laws in self-healing properties by fatigue-healing-fatigue test (intermediate temperature cracking) and fracture-healing-fracture test (low temperature cracking). The main research goals include (1) exploring the self-healing properties of different types of asphalt after secondary aging process; (2) after secondary aging, observing whether the asphalt still has the potential to be healed; (3) recording the self-healing properties of recycled asphalt under different conditions. The research results of this paper can enrich the findings about the change laws in the self-healing performance of different types of asphalt mastics after secondary aging and promote the understanding of multiple recycling applications of RAP.

## 2. Raw Materials and Methodologies

### 2.1. Materials

#### 2.1.1. Raw Materials

The new asphalts used in this study mainly include petroleum asphalt with the penetration grade of 70# and SBS-modified asphalt with the PG grade of 76-22. The performance indicators of the asphalts are shown in Table 1 and Table 2.

#### 2.1.2. Extracted Old Asphalt from RAP

The RAP materials used in this study originated from the Suzhou section of the Nanjing-Shanghai expressway, which suffered 6 years of service life. According to the standard methods in JTG E20-2011 (T0722), the old asphalt was extracted from RAP, and the corresponding properties were tested and summarized in Table 3. It can be seen from Table 3 that the penetration of the extracted old asphalt still reaches 34 (0.1 mm), which meets the requirements for hot in-place recycling according to the JTG/T 5521-2019 [30].

#### 2.1.3. New Fillers

Limestone mineral powder was selected as the filler and the specific technical indicators are shown in Table 4.

#### 2.1.4. Rejuvenating Agent

The rejuvenating agent used in this study is Runqiang-RA102 high-performance asphalt rejuvenator produced by Jiangsu Subote Company (Nanjing, China). The specific technical indicators are shown in Table 5.

### 2.2. Preparation of Asphalt Mastic Samples

#### 2.2.1. Asphalt Aging Process and Asphalt Mastic Fabrication

**Figure 1:**
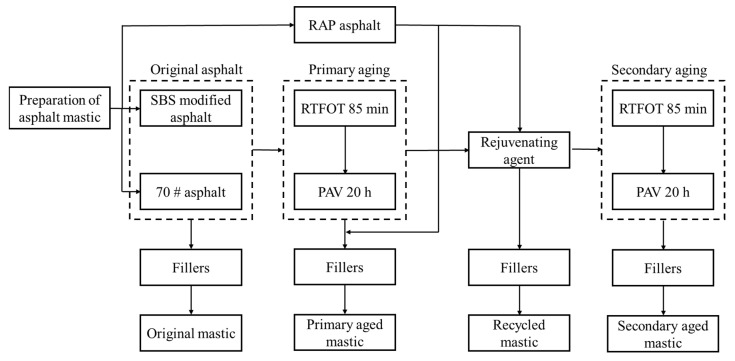
Preparation procedures of asphalt mastics.

The asphalt mastic samples were prepared according to the following steps. The overall mastic preparation scheme is shown in Figure 1.

(1) Original mastic samples were prepared by adding mineral powders into the new 70# asphalt and SBS-modified asphalt.
(2) Primary aged mastic samples were prepared by adding mineral powders into the aged asphalts and the extracted old asphalt from RAP. The aged 70# asphalt and SBS-modified asphalts were artificially made by rolling thin-film oven test (RTFOT) and pressurized aging vessel (PAV) test.

Recycled mastic samples were prepared by adding the rejuvenating agent into the primary aged asphalts and extracted old asphalt, and then mixed with mineral powders. 

Secondary aged mastics were prepared by adding mineral powders into the secondary aged asphalts by RTFOT short-term and PAV long-term aging. 

Besides, the mixing procedures for the asphalt mastics were showing as the following steps. Firstly, asphalt and mineral powders were weighed and preheated. Then, the preheated asphalt was put on a heating furnace to maintain a temperature of 155–165 °C. Thirdly, mineral powders were added into the asphalt continuously, and the asphalt mastic was stirred at a speed of 2000 r/min for 20 min to make it homogeneous.

#### 2.2.2. Rejuvenating Agent Dosing

The recycled asphalt samples were prepared by mixing the aged asphalt with a rejuvenating agent. Four dosage levels of 2%, 3%, 4%, and 6% were set to conduct these tests, by weight of the aged asphalt. Then, the needle penetration, softening point, and viscosity were measured, and the results are listed in Table 6, Table 7 and Table 8. It can be observed from Table 6, Table 7 and Table 8 that the indexes of all three types of asphalt recovered to an acceptable level when 6% of rejuvenating agent was used. In order to avoid the negative impact caused by excessive rejuvenating agent, 6% of rejuvenating agent was selected for this study.

### 2.3. Fatigue-Healing-Fatigue Test Method

#### 2.3.1. Experimental Parameters

The fatigue-healing-fatigue test was carried out by DSR time scanning. The diameter of the parallel plate of 8 mm and the spacing of 2 mm were set. The test temperature of 25 °C, loading frequency of 10 Hz, and 4% of the fatigue loading strain were applied. The healing temperatures for 70# asphalt were set to be 25 °C, 35 °C, and 45 °C, while the healing temperatures for SBS-modified asphalt and RAP extracted old asphalt were 25 °C, 45 °C, and 65 °C. The healing times for all the three types of mastics were 10 min, 20 min, 30 min, 40 min, 50 min, and 70 min. Three duplicates were used for each test condition. In addition, the timing when the selected complex modulus dropped to 50% of the initial value was chosen as the termination point to simulate 50% fatigue damage degree of the samples. The specific test parameters are summarized in Table 9.

#### 2.3.2. Evaluating Indicator

**Figure 2:**
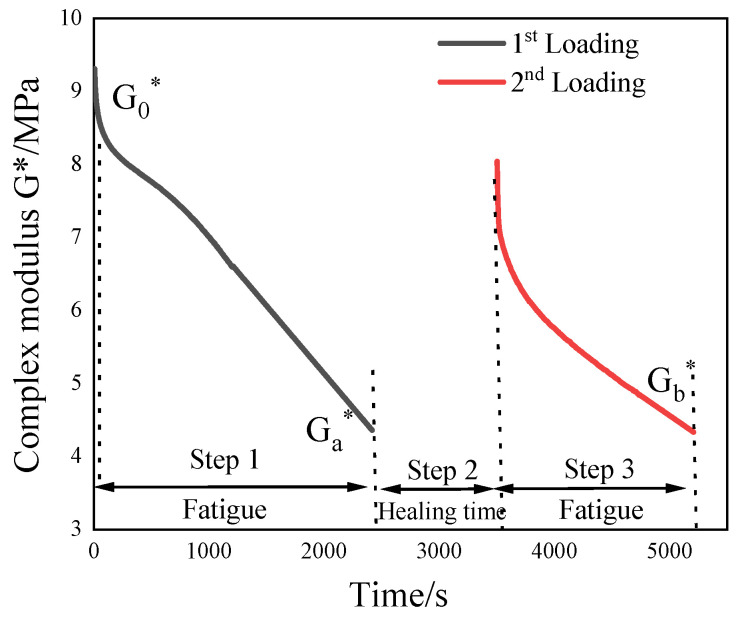
Schematic diagram of F-H-F test.

In this study, the cumulative dissipated energy ratio of asphalt mastic before and after healing was used as the evaluation index of fatigue healing performance. Figure 2 is the schematic diagram of the test, and the calculation processes are shown in Equation (1) to Equation (3).

(1) $$W_{i}=\pi \sigma \varepsilon \sin(\delta )$$

(2) $$W=\sum W_{i}$$

(3) $${\mathrm{HI}}_{1}=Wa/Wb$$

where *HI*_1_ is the healing index, %; *W* is dissipation energy, MJ/m^3^; *σ* is the stress amplitude, MPa; *ε* is the applied strain, %; *δ* is the phase angle, °; *W_b_* and *W_a_* are the cumulative dissipated energy before and after the healing process of asphalt mastic, MJ/m^3^.

### 2.4. Fracture-Healing-Fracture Test

Based on direct tension test (DTT), the fracture-healing-fracture test was employed to simulate the brittle fracture process of asphalt materials under low temperature conditions and recovery process after a period of healing time at certain temperatures [32,33].

#### 2.4.1. Specimen Preparation

**Figure 3:**
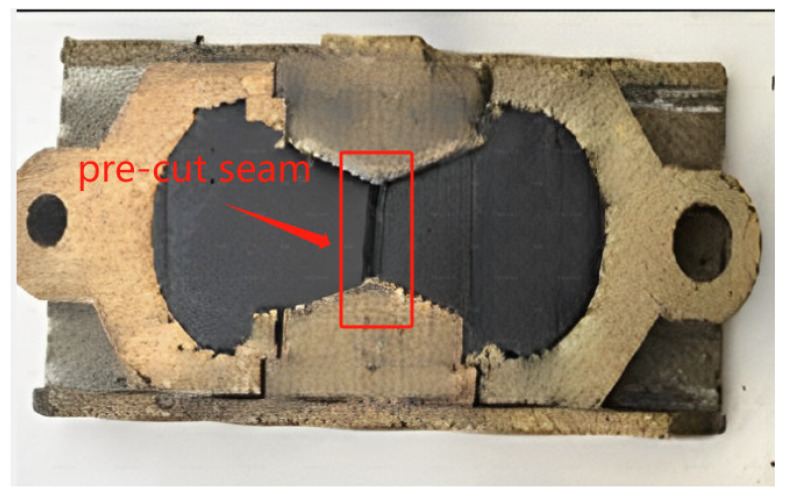
Illustration of an asphalt mastic specimen for fracture-healing-fracture test.

The fracture-healing-fracture specimens were prepared according to the requirements of T0629 method in JTG E20, which is similar to the specimens for ductility test. In order to ensure that the specimens break at the middle position during the direct tensile process, a pre-cut seam with the width of 2 mm and depth of 1 mm was artificially made in the middle of the specimens, as shown in Figure 3.

#### 2.4.2. Experimental Parameters and Evaluating Indicator

The test temperature was set to be −10 °C. The tensile rate was 100 mm/min, and the test was terminated after the specimen was broken. In order to ensure the full healing of asphalt mastic, the healing time was determined to be 4 h, 8 h, and 12 h, respectively. Three duplicates were used for each test condition. The healing temperatures were the same as those of fatigue-healing-fatigue test. The specific test parameters are shown in Table 10.

**Figure 4:**
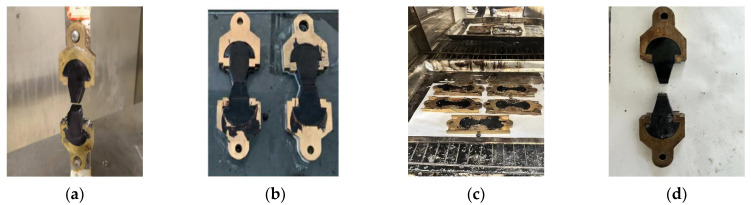
Fracture-healing-fracture test procedures. (**a**) first tensile; (**b**) bonding; (**c**) healing; (**d**) secondary tensile.

According to the load-displacement curve, the peak stress σ_0_ corresponding to the fracture of asphalt mastic can be used to evaluate its low temperature crack resistance. After the primary tensile fracture, the specimen was immediately placed in the module and kept warm at the targeted healing temperature. After a certain healing time, the tensile test was carried out again to obtain the peak stress *σ*_1_ after healing. The ratio of the two stresses after and before the healing process was defined as the fracture healing index *HI*_2_. The specific test processes are shown in Figure 4. The calculation formula is shown in Formula (4).

(4) $${\mathrm{HI}}_{2}=\sigma 1/\sigma 0$$

In the formula: *σ*_0_ is the peak value of the first tensile fracture stress, MPa; *σ*_1_ is the peak value of the secondary tensile fracture stress, MPa.

## 3. Results and Discussions

### 3.1. Fatigue-Healing-Fatigue Test Results

**Figure 5:**
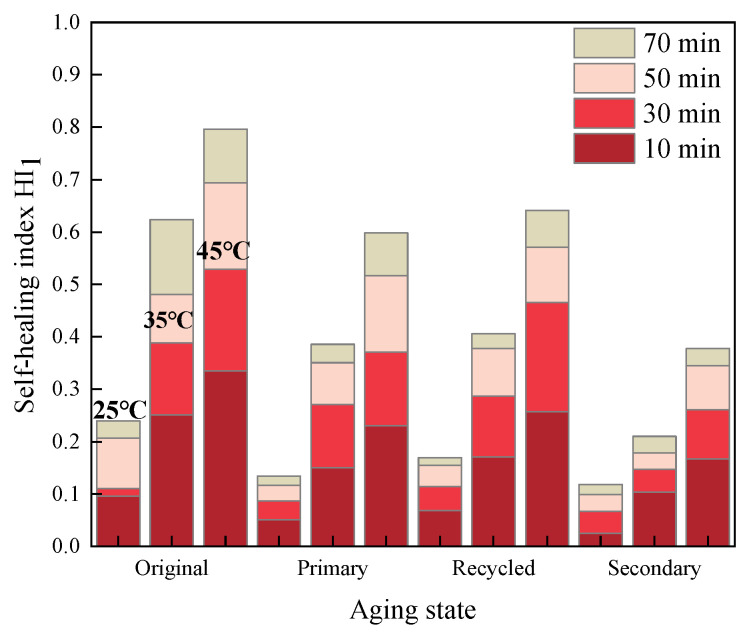
*HI*_1_ of 70# asphalt mastics.

**Figure 6:**
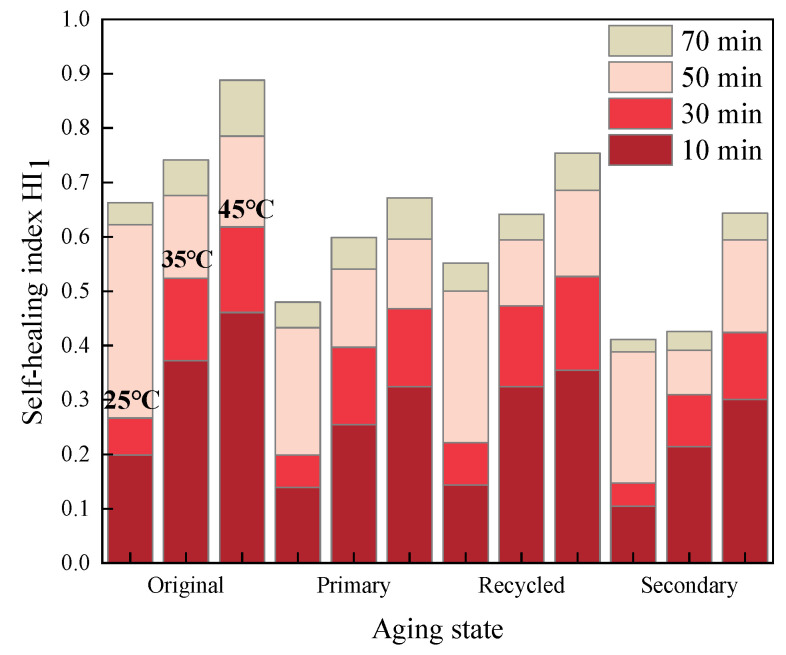
*HI*_1_ of SBS-modified asphalt mastics.

**Figure 7:**
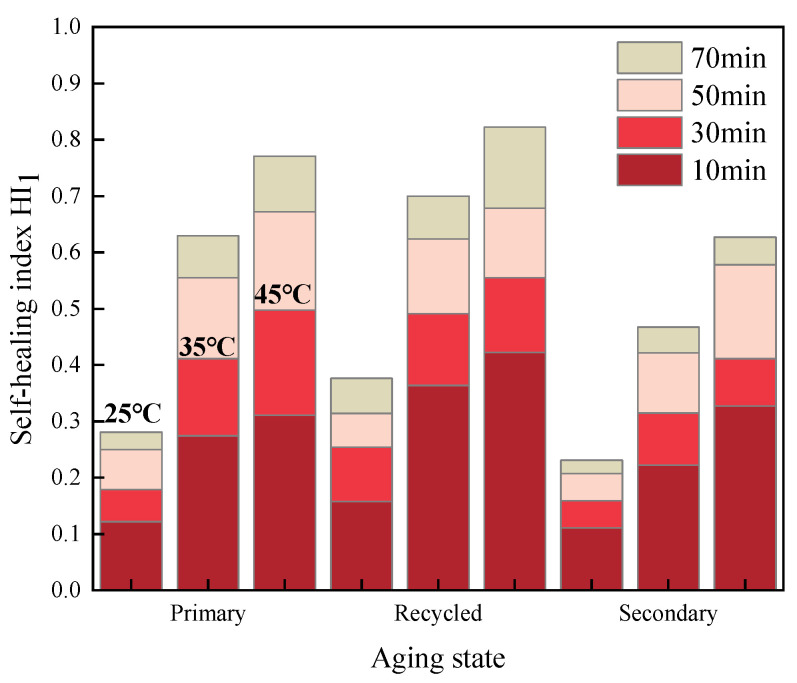
*HI*_1_ of extracted old asphalt mastics.

The healing index values for fatigue-healing-fatigue tests of various asphalt mastics under different test conditions were calculated and recorded as *HI*_1_. The specific results are illustrated in Figure 5, Figure 6 and Figure 7.

Comparing the results of Figure 5, Figure 6 and Figure 7, it becomes evident that within a certain range, a higher healing temperature, a longer healing time, and the addition of a rejuvenating agent can contribute to enhancing the fatigue healing properties of asphalt mastics. Furthermore, deepening the aging degree will lead to a deterioration in the healing performance to some extent.

#### 3.1.1. Effect of Healing Time on Fatigue Self-Healing Properties

**Figure 8:**
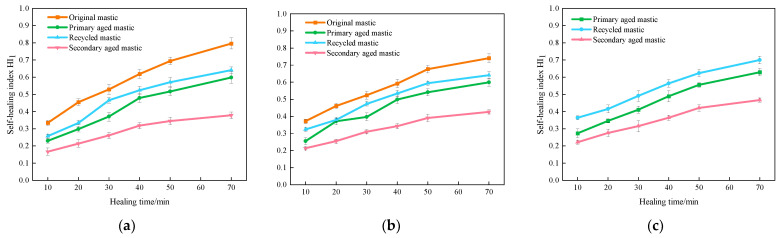
Results of self-healing index *HI*_1_ of asphalt mastics at different healing times. (**a**) *HI*_1_ of 70# asphalt mastics at 45 °C; (**b**) *HI*_1_ of SBS-modified asphalt mastics at 45 °C; (**c**) *HI*_1_ of extracted old asphalt mastics at 45 °C.

Taking the results from the test temperature of 45 °C as examples, the *HI*_1_ results are shown in Figure 8.

It can be seen from Figure 8 that the fatigue healing index *HI*_1_ values of all kinds of asphalt mastics, despite the aging states, increase with an increasing healing time, indicating that the self-healing performance is gradually recovering. For the unaged original asphalt mastics, the fatigue healing index *HI*_1_ of 70# asphalt mastic reaches 0.335, 0.529, 0.694, and 0.796, respectively, at the healing timing of 10 min, 30 min, 50 min, and 70 min. Meanwhile, the corresponding HI_1_ value of SBS-modified asphalt mastic is 0.372, 0.524, 0.676, and 0.741, respectively. It infers that the unaged 70# and SBS-modified asphalt mastics present comparable healing ability after fatigue damage.

After primary aging, the *HI*_1_ values of all kinds of asphalt mastics reduce significantly. The *HI*_1_ values of primary aged 70# asphalt mastics decrease from 65.5% to 77.4% of the unaged original mastics, at healing timing from 10 min to 70 min. Meanwhile, the corresponding *HI*_1_ values of primary aged SBS-modified asphalt mastics reduce from 68.5% to 84.1% of the original ones. It can be noticed that with healing time, the healing capability of primary aged mastics tends to approach the original ones. After adding rejuvenating agent, the *HI*_1_ values of recycled mastics recover to some extent, but cannot reach the same level as the original mastics.

After secondary aging, the HI_1_ values of all kinds of asphalt mastics further reduce. The HI_1_ values of secondary aged 70# asphalt mastics are around 50% of the corresponding original unaged ones, while the HI_1_ values of secondary aged SBS-modified asphalt mastics decreases to 65.3–75.7% of the original ones, at the healing timing of 10–70 min. In addition, the *HI*_1_ values of extracted old asphalt mastics are close to those of SBS-modified asphalt mastics at the same test condition. Generally speaking, after secondary aging, 70# asphalt mastics show the worst healing capability, while SBS-modified asphalt and extracted old asphalt presenting the similar superior healing ability, indicating that 70# asphalt being more sensitive to secondary aging process [34].

#### 3.1.2. Effect of Healing Temperature on Fatigue Self-Healing Properties

**Figure 9:**
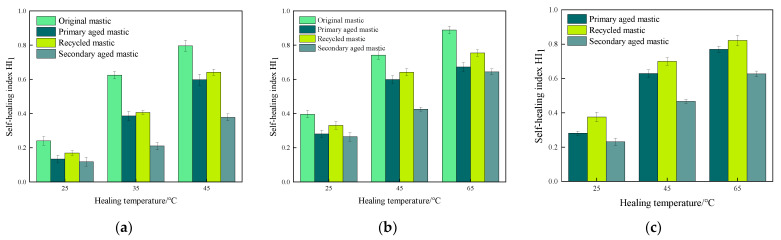
The results of *HI*_1_ of asphalt mastics at different healing temperatures. (**a**) *HI*_1_ of 70# asphalt mastic at 70 min; (**b**) *HI*_1_ of SBS-modified asphalt mastic at 70 min; (**c**) *HI*_1_ of extracted old asphalt mastics at 70 min.

Taking the results from the healing time of 70 min as examples, the *HI*_1_ results are illustrated in Figure 9.

It can be seen from Figure 9 that the healing index *HI*_1_ is positively correlated with the temperature. As the healing temperature increases, the *HI*_1_ values of the asphalt mastics increase to a great extent. In terms of original asphalt mastics, the *HI*_1_ value of 70# asphalt mastic increases by 232% at 45 °C than that at 25 °C, while the *HI*_1_value of SBS-modified asphalt increases by 87.1%. It infers that the self-healing characteristic of 70# asphalt mastics is more sensitive to temperatures than that of SBS-modified asphalt mastics [35].

After primary aging, the self-healing performance of all types of asphalt mastics deteriorates. At the healing temperatures of 25 °C, 35 °C, and 45 °C, the *HI*_1_ values of primary aged 70# asphalt mastics decrease to 55.8%, 61.9%, and 75.1% of the original unaged ones, respectively. Meanwhile, at the healing temperatures of 25 °C, 45 °C, and 65 °C, the HI_1_ values of primary aged SBS-modified asphalt mastics reduce to 71.0%, 80.8%, and 75.7% of the original ones, respectively. It reflects that the self-healing ability of primary aged asphalt mastics is approaching the original asphalt mastics with an increasing healing temperature but cannot reach the same level, indicating primary aging causes some damage to the fatigue healing characteristics of asphalt mastics [36].

After adding rejuvenating agent, the self-healing performance of recycled asphalt mastics was recovered to some certain extent. The fatigue healing index *HI*_1_ values of recycled 70#, SBS, and old asphalt mastics increase to 107.2%, 107.0%, and 111.3% of that of corresponding primary aged mastics at 45 °C.

After secondary aging, the healing index *HI*_1_values of all mastics still increase with an increasing healing temperature. However, some differences can be found in the *HI*_1_ values of different mastics. At the healing temperatures of 25 °C, 35 °C, and 45 °C, the HI_1_ values of secondary aged 70# asphalt mastics reduce to 69.8%, 51.7%, and 59.0% of the recycled mastics, respectively. Meanwhile, the *HI*_1_ values of secondary aged SBS-modified asphalt mastics decrease to 79.8%, 66.5%, and 85.4% of the corresponding recycled ones, respectively, while the extracted old mastics presenting similar *HI*_1_ values to secondary aged SBS asphalt mastics. This suggests that after secondary aging, the healing ability of 70# asphalt mastics can hardly recover to a satisfactory level compared to that of primary aged ones, even at a higher healing temperature. However, the healing ability of SBS-modified asphalt mastics still shows the comparable level to that of primary aged ones. These findings also can indicate that SBS-modified asphalt have better anti-aging capability than 70# asphalt, resulting in superior healing performance [35].

#### 3.1.3. Effect of Aging State of Asphalt on Fatigue Self-Healing Properties

**Figure 10:**
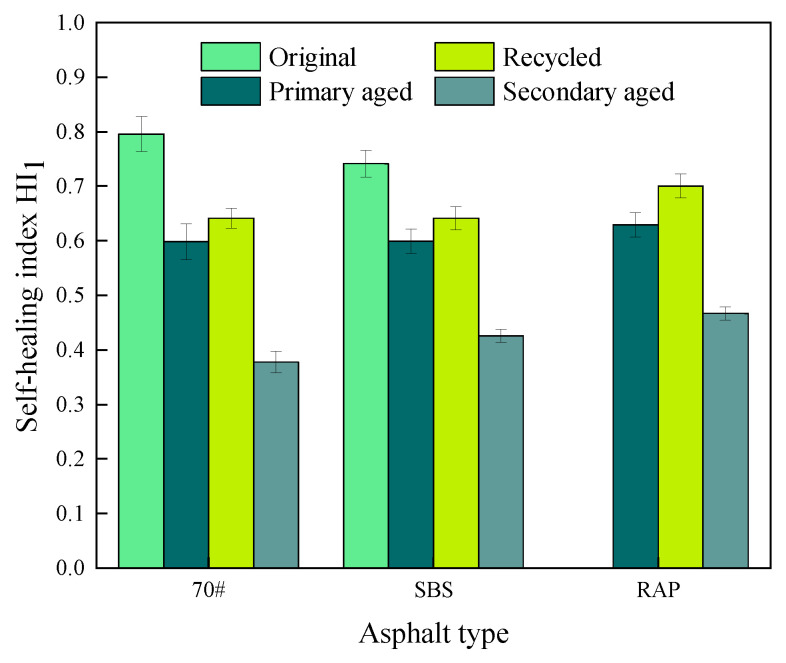
Results of *HI*_1_ of asphalt mastics at different aging states.

Taking the *HI*_1_ results at the healing temperature of 45 °C and the healing time of 70 min as examples, the effect of aging state of asphalt on the of self-healing index *HI*_1_ of 70# asphalt, SBS-modified asphalt, and extracted old asphalt mastics are shown in Figure 10.

It can be seen from Figure 10 that aging state has great impact on the healing properties of asphalt mastics. In terms of 70# asphalt mastics, the *HI*_1_ values reduce to 75.1%, 80.5%, and 47.5% of the original unaged mastics after primary aging, recycling process, and secondary aging, respectively. As for SBS-modified asphalt mastics, the corresponding *HI*_1_ values decrease to 80.8%, 86.5%, and 57.5%, respectively. This suggest that the deeper the aged degree achieves, the worse healing ability of the mastics will be, though the rejuvenating agent can recover the healing ability to some degree. In summary, as for the original asphalt mastics, the self-healing properties of 70# asphalt mastics are superior to those of SBS-modified asphalt mastic. After primary aging and secondary aging, the healing ability of SBS-modified asphalt mastics becomes better than that of 70# asphalt mastics. This is mainly because the SBS modifier absorbs some light oils of the matrix asphalt and swells to form a spatial three-dimensional network structure, which improves the viscoelastic properties of the asphalt after the aging process [37]. After a period of healing time, its complex modulus can restore to a large value, thus presenting good healing ability [38].

### 3.2. Fracture-Healing-Fracture Test Results

**Figure 11:**
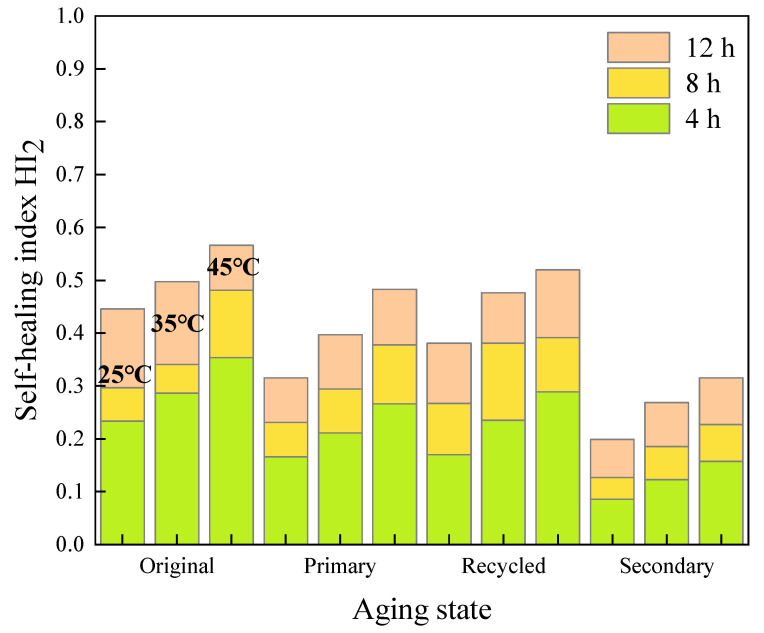
*HI*_2_ of 70# asphalt mastics.

**Figure 12:**
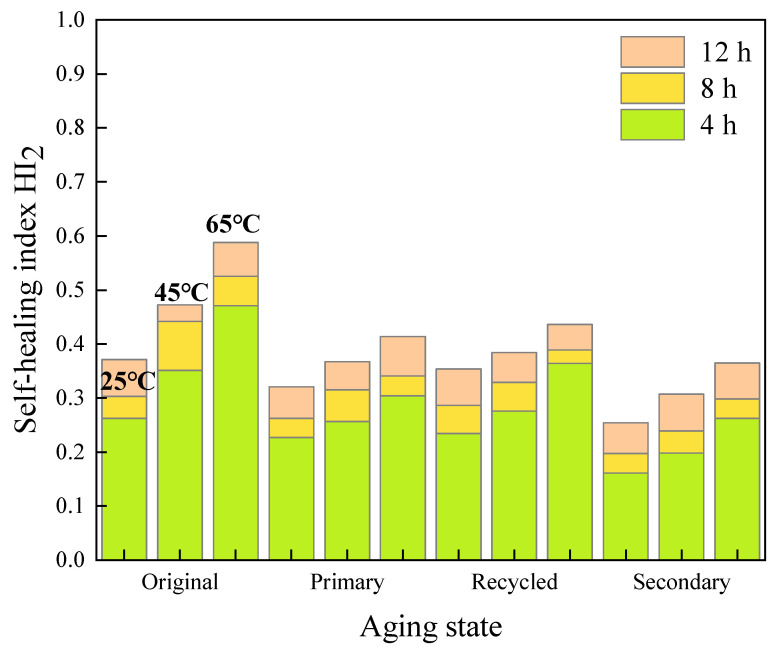
*HI*_2_ of SBS-modified asphalt mastics.

**Figure 13:**
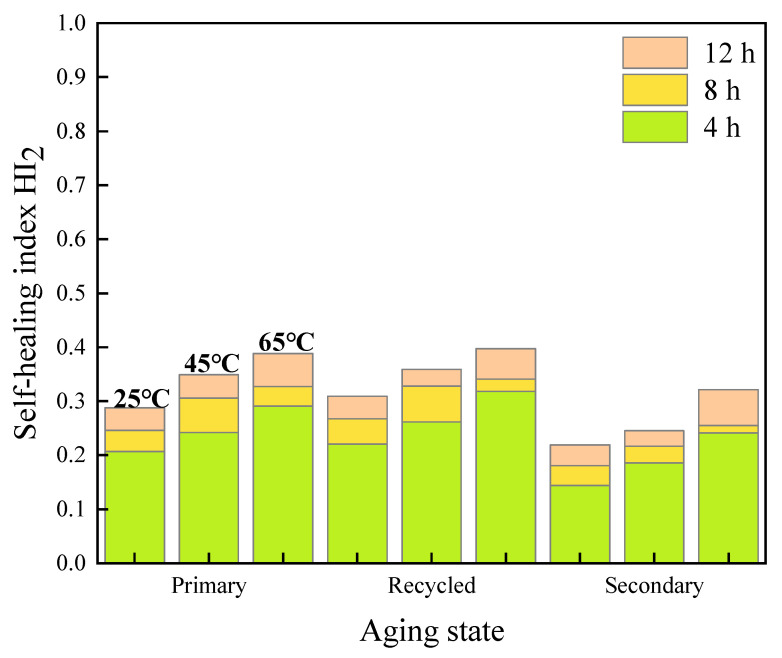
*HI*_2_ of extracted old asphalt mastics.

The fracture healing index *HI*_2_ values of various asphalt mastics under different test conditions were calculated, and are illustrated in Figure 11, Figure 12 and Figure 13.

It can be seen from the experimental results that the change laws of fracture healing index are similar to that of fatigue healing ones. The healing time, healing temperature, and rejuvenating agent are positively correlated with healing index *HI*_2_, with aging degree also leading to a negative correlation with *HI*_2_.

#### 3.2.1. Effect of Healing Time on Fracture Self-Healing Properties

**Figure 14:**
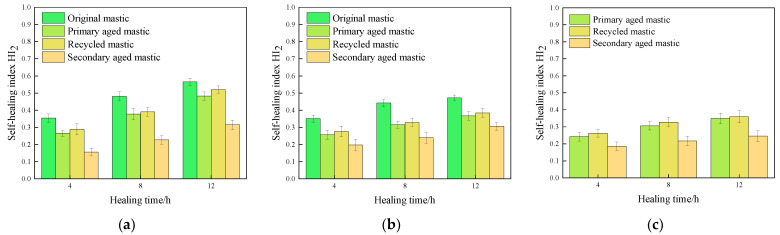
Results of self-healing index *HI*_2_ of asphalt mastics at different healing time. (**a**) *HI*_2_ of 70# asphalt mastics 45 °C; (**b**) *HI*_2_ of SBS-modified asphalt mastics at 45 °C; (**c**) *HI*_2_ of extracted old asphalt mastics at 45 °C.

The results of the test at temperature of 45 °C are used and illustrated in Figure 14.

In terms of the original asphalt mastics, the *HI*_2_ values of 70# asphalt mastics increase to 136% and 159% after curing for 8 h and 12 h, respectively, compared to those of after 4 h healing. Meanwhile, the corresponding *HI*_2_ values of SBS-modified asphalt mastics reach to 126% and 134% of those after 4 h healing, respectively.

After primary aging, the *HI*_2_ of 70# asphalt mastic presents the highest value at different healing time. After curing for 12 h, the fracture healing index *HI*_2_ reach to 182%, 143% and 144% of those after 4 h healing, for 70# asphalt, SBS-modified, and extracted old asphalt mastics, respectively.

After secondary aging, the *HI*_2_ values of all kinds of asphalt mastic decrease significantly, but still increases with an increasing healing time. After curing for 12 h, the *HI*_2_ values reach to 201%, 155%, and 132% of the corresponding asphalt mastics after 4 h healing, for the 70# asphalt, SBS-modified, and extracted old asphalt mastics, respectively. It is worth pointing out that the absolute values of *HI*_2_ still maintain at low levels of less than 0.5 for the primary aged mastics and around 0.3 for the secondary aged ones. In terms of the extracted old asphalt mastic, the *HI*_2_ values range from 0.185 to 0.245 after secondary aging, presenting the weakest fracture healing ability.

#### 3.2.2. Effect of Healing Temperature on Fracture Self-Healing Properties

**Figure 15:**
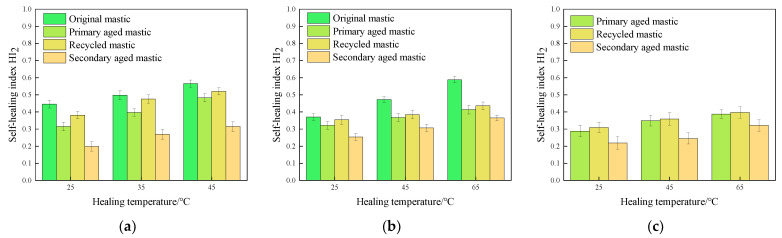
Results of self-healing index *HI*_2_ of asphalt mastics at different healing temperatures. (**a**) *HI*_2_ of 70# asphalt mastics after 12 h healing; (**b**) *HI*_2_ of SBS-modified asphalt mastics after 12 h healing; (**c**) *HI*_2_ of extracted old asphalt mastics after 12 h healing.

The results of the tests after 12 h healing are used and illustrated in Figure 15.

It can be seen from Figure 15 that the *HI*_2_ values of all kinds of asphalt mastics increase with the increase of temperature. As for the unaged original asphalt mastics, after healing 12 h at 35 °C and 45 °C, the *HI*_2_ values of 70# asphalt mastics reached to 111% and 127% of the corresponding mastics healing at 25 °C, respectively. In terms of SBS-modified asphalt mastics, after healing 12 h at 45 °C and 65 °C, the *HI*_2_ values reached to 127% and 158% of the corresponding mastics at 25 °C, respectively. In addition, the *HI*_2_ value of SBS-modified asphalt mastics increases significantly at 65 °C, achieving a higher absolute value than the *HI*_2_ value of 70# asphalt mastics at 45 °C. It indicates that SBS-modified asphalt mastics need higher healing temperature than that of 70# asphalt mastics.

After primary aging, the *HI*_2_ values of 70# asphalt mastics at 25 °C, 35 °C, and 45 °C decrease to 70.6%, 79.9%, and 85.3% of the original unaged asphalt mastics, respectively. At the healing temperatures of 25 °C, 45 °C, and 65 °C, the *HI*_2_ values of SBS-modified asphalt mastic reduce to 86.5%, 77.7%, and 70.4% of the original asphalt mastics, respectively. It can be seen that the primary aged 70# asphalt mastics show superior fracture healing ability than that of SBS-modified asphalt mastics.

After secondary aging, the *HI*_2_ values of all kinds of asphalt mastics decrease significantly, of which the 70# asphalt mastics show the most obvious drop. The *HI*_2_ values of secondary aged 70# asphalt mastics reduce to 44.6%, 54.1%, and 55.7% of the original unaged asphalt mastics, respectively. Meanwhile, the *HI*_2_ values of the secondary aged SBS-modified asphalt mastics decrease to 68.5%, 65.0%, and 62.1% of the original ones, respectively. It could be noticed that the recovery degree of both secondary aged 70# and SBS-modified asphalt mastics can hardly increase with an increasing healing temperature, though the absolute *HI*_2_ value shows a rising trend with temperature.

#### 3.2.3. Effect of Aging State of Asphalt on Fracture Self-Healing Properties

**Figure 16:**
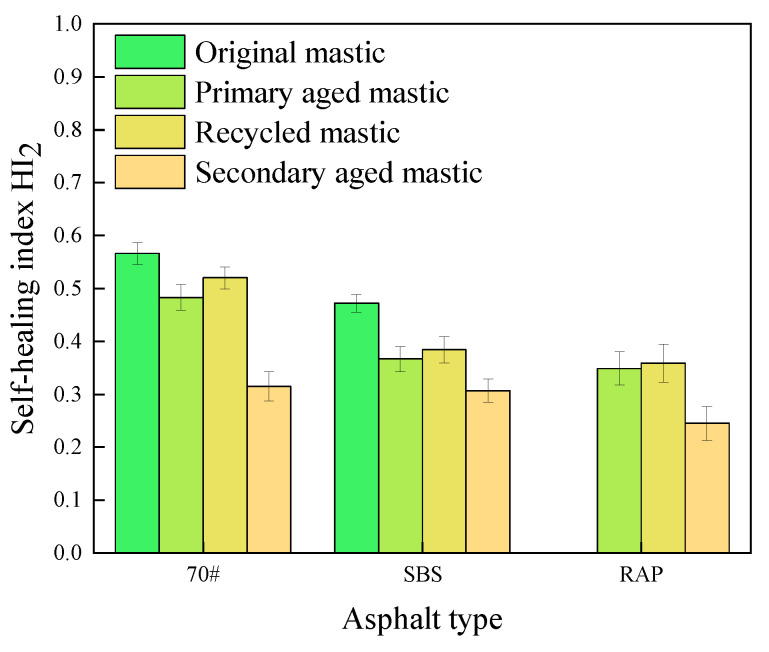
Results of self-healing index *HI*_2_ of asphalt mastics in different aging states.

The results of the test at temperature of 45 °C and healing time of 12 h are used and illustrated in Figure 16.

It can be seen from Figure 16 that as for the original unaged asphalt mastic, the *HI*_2_ value of 70# asphalt mastic is higher than that of SBS-modified asphalt mastic, indicating that 70# asphalt mastics have a better fracture healing ability. After primary aging, the *HI*_2_ value of 70# asphalt mastic decreases from 0.566 to 0.483, which reduced to 85.3% of the original mastic. The *HI*_2_ value of SBS-modified asphalt mastic decreases from 0.472 to 0.367, which reduced to 77.8% of the original one. This indicates that the aging process has a more significant effect on the degradation of self-healing performance of SBS-modified asphalt mastic, which is also related to the slow diffusion behavior of SBS modifier [39].

After adding a rejuvenating agent, the *HI*_2_ of recycled 70# asphalt mastic achieves 91.9% of the original mastic, and the index of SBS-modified asphalt mastic achieved 81.4% of the original mastic. The index of recycled old asphalt mastic is also improved compared to the extracted old asphalt mastic.

After secondary aging, the self-healing ability of all kinds of asphalt mastics deteriorate more seriously than that of the primary aged mastics. The *HI*_2_ values of 70#, SBS, and old asphalt mastics decrease to 60.6%, 79.9%, and 68.2% of the corresponding recycled mastics, respectively.

Overall, during the fracture-healing-fracture test, the asphalt mastics suffered a full fracture. Due to the SBS modifier’s interconnecting structure in asphalt, it is difficult for the SBS molecules to diffuse and mix with each other on the two fracture sections during the healing process, resulting in a lower fracture healing index of SBS and old asphalt mastics. Furthermore, the performance of secondary aged SBS-modified asphalt mastics is approaching that of 70# asphalt mastics. It can be partly attributed to the decomposition of SBS modifier [40], resulting in the close fracture healing index values of different mastics.

### 3.3. Comprehensive Analysis

**Figure 17:**
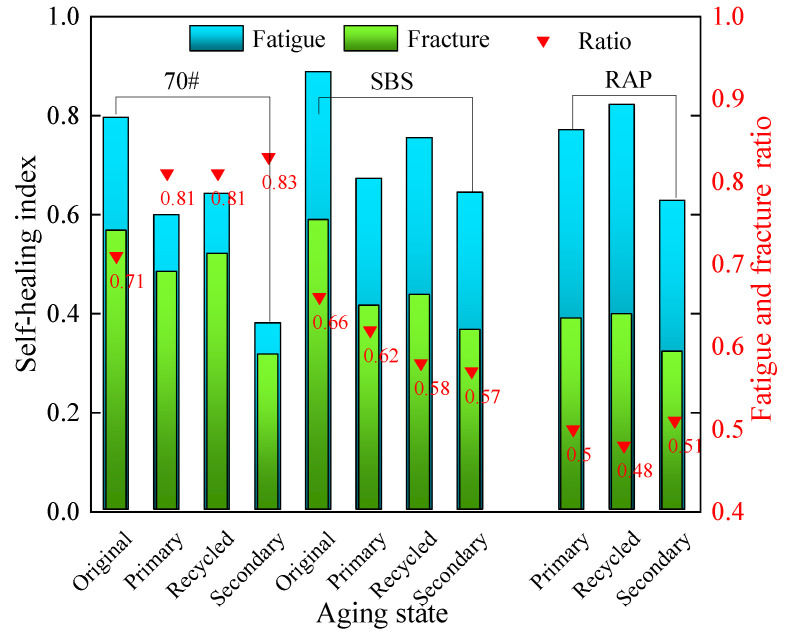
Comparison of fatigue healing and fracture healing indexes.

The highest index values of both fatigue healing and fracture healing of different asphalt mastics were chosen for this comprehensive analysis, and the results are illustrated in Figure 17.

From Figure 17, it can be seen that the fracture healing ability of the three types of asphalt mastics is much inferior to the fatigue healing performance, indicating that low-temperature cracks being hardly cured by self-healing rather than fatigue cracks.

In terms of 70# asphalt mastics, the ratios of *HI*_2_ over *HI*_1_ reach 71.1%, 80.8%, 81.1% and 83.3% for the original unaged, primary aged, recycled, and secondary aged mastics, respectively. It infers that the fatigue healing ability and fracture healing ability of 70# asphalt mastics are comparable. However, the corresponding ratios only achieve 66.2%, 61.6%, 57.8%, and 56.7% for SBS-modified asphalt mastics. The extracted old asphalt mastics also display small ratio values similar to SBS-modified asphalt mastics. These results indicate that the fatigue and fracture healing properties depend on asphalt type, and 70# asphalt mastics present similar fatigue and fracture healing properties, while SBS-modified asphalt mastics possessing excellent fatigue but insufficient fracture healing properties. The reason can be that SBS modifier can improve the viscoelastic property of asphalt, resulting in excellent fatigue healing ability of SBS-modified asphalt. However, the macromolecule of the SBS modifier can hardly diffuse though the fracture section, leading to inferior fracture healing property.

## 4. Conclusions

This article used fatigue-healing-fatigue test and fracture-healing-fracture test to investigate the self-healing performance of 70# asphalt, SBS-modified asphalt, and RAP extracted old asphalt mastics under different healing times, healing temperatures, and aging states fabricated by primary and (or) secondary aging processes. The cumulative dissipation energy ratio and tensile fracture stress ratio of asphalt mastics after and before healing was used as fatigue healing index and fracture healing index, respectively. Under the testing conditions mentioned above, the following conclusions can be drawn:

(1) With an increasing healing time and temperature, the fatigue healing index (*HI*_1_) values increase up to around 0.8 and 0.9 for unaged 70# asphalt and SBS-modified asphalt mastics, respectively. The absolute values of fracture healing index (*HI*_1_) values reach just around 0.57 and 0.47. It means both of these two mastics present comparably excellent fatigue healing and inferior fracture healing ability.
(2) Aging state has significant impact on the healing property of asphalt mastics. After primary and secondary aging, the fatigue healing index (*HI*_1_) values reduce from 0.796 of unaged 70# asphalt mastics to 0.598 and 0.378, while the *HI*_1_ values of SBS-modified asphalt mastics reduce from 0.888 of unaged one to 0.672 and 0.674, respectively.
(3) Healing properties are also asphalt-type dependent, and 70# asphalt is more sensitive to secondary aging process. After secondary aging, 70# asphalt mastics show the worst fatigue healing capability and can hardly recover to a satisfactory level, while SBS-modified asphalt and extracted old asphalt presenting the similar superior healing ability to that of primary aged ones.
(4) Low-temperature cracks of aged asphalts can hardly be cured by self-healing rather than fatigue cracks. After primary and secondary aging, the fracture healing index (*HI*_2_) of all the three types of mastics are only around 0.3, which are much smaller than the *HI*_1_ values under each testing condition, and can hardly increase with an increasing healing temperature.
(5) The recover effect of the rejuvenating agent will be degraded rapidly during the secondary aging process, though both the fatigue and fracture healing performance could be recovered to a certain extent by adding rejuvenating agent. Thus, more effective rejuvenating agents are on demand to be developed.

This study just explored the self-healing performance of asphalt mastics by experiments. There are several points that can be considered for further investigation. Firstly, microstructure and chemical characteristics of asphalt mastics after aging and recycling processes can be investigated by scanning electron microscopy (SEM) and atomic force microscopy (AFM), etc., and the relationships with the self-healing properties can be established. Secondly, only one-time damage was utilized in this study. The effect of multiple damages on the self-healing performance of asphalt mastics also needs further investigation. Thirdly, the effect of secondary aging on the road performance and self-healing properties of recycled asphalt mixtures is also worth studying, in addition to the results of asphalt mastics. 

## Figures and Tables

**Table 1:** Performance indicators and test results of 70# asphalt.

Index	Requirements in JTG-F40 [28]	Results	Test Method in JTG-E20 [29]
Penetration value (25 °C, 0.1 mm)	60~80	68	T0604
Softening point (°C)	≥45	49	T0606
Ductility (5 cm/min, 15 °C, cm)	≥100	165.1	T0605
Flashpoint (°C)	≥260	321	T0661
Viscosity at 60 °C (Pa·s)	≥180	213	T0604

**Table 2:** Performance index and test results of SBS-modified asphalt.

Index	Requirements in JTG-F40 [28]	Results	Test Method in JTG-E20 [29]
Penetration value (25 °C, 0.1 mm)	30~60	56	T0604
Softening point (°C)	≥60	82	T0606
Ductility (5 cm/min, 5 °C, cm)	≥20	38	T0605
Separation (°C)	≤2.5	1.4	T0661
Elastic recovery (25 °C, %)	≥75	76	T0662

**Table 3:** Performance index and test results of extracted old asphalt.

Index	New SBS-Modified Asphalt	Extracted Old Asphalt	Test Method in JTG-E20 [29]
Penetration value (25 °C, 0.1 mm)	56	34	T0604
Softening point (°C)	82	66	T0606
Ductility (5 cm/min, 5 °C, cm)	38	8.3	T0605
Viscosity at 135 °C (Pa·s)	2.35	3.46	T0613

**Table 4:** Performance indexes and test results of mineral powder.

Index		Requirements in JTG-E42 [31]	Results	Test Method in JTG-E42 [31]
Water content (%)		≤1.0	0.3	Oven dry
Hydrophilic coefficient		<1.0	0.63	T0353
Plasticity index (%)		<4.0	2.5	T0354
Passing (%)	<0.6 mm	100	100	T0351
	<0.15 mm	92.6	90–100	
	<0.075 mm	92.2	75–100	

**Table 5:** Performance indexes of rejuvenating agent.

Index	Requirements in JTG/T 5521 [30]	RA-102	Test Method in JTG-E20 [29]
Viscosity at 90 °C (cP)	-	4000	T0619
Flashpoint (°C)	≥220	248	T0633
Saturated hydrocarbons content (%)	≤30	25.6	T0618
Aromatic content (%)	≥30	53	T0618
Mass loss after RTFOT (%)	≤4%	1.02	T0603

**Table 6:** Physical properties of recycled 70# asphalt.

Index	Original	Rejuvenating Agent Content (%)					Test Method in JTG-E20 [29]
		0	2	4	6	8	
Penetration value (25 °C, 0.1 mm)	68.2	30.7	43.8	52.4	61.2	68.1	T0604
Softening point (°C)	48.5	62.2	57.3	54.5	50.7	46.3	T0606
Viscosity at 135 °C (Pa·s)	0.54	1.01	0.94	0.83	0.66	0.46	T0613

**Table 7:** Physical properties of recycled SBS-modified asphalt.

Index	Original	Rejuvenating Agent Content (%)					Test Method in JTG-E20 [29]
		0	2	4	6	8	
Penetration value (25 °C, 0.1 mm)	58.0	31.0	38.4	45.2	51.4	57.8	T0604
Softening point (°C)	68.2	76.2	72.1	68.6	66.3	61.5	T0606
Viscosity at 135 °C (Pa·s)	2.89	4.75	4.33	3.51	2.88	2.54	T0613

**Table 8:** Physical properties of recycled RAP old asphalt.

Index	Rejuvenating Agent Content (%)					Test Method in JTG-E20 [29]
	0	2	4	6	8	
Penetration value (25 °C, 0.1 mm)	34.1	39.2	44.4	52.7	56.2	T0604
Softening point (°C)	66.3	63.7	61.6	59.1	54.0	T0606
Viscosity at 135 °C (Pa·s)	3.46	3.15	2.98	2.64	2.17	T0613

**Table 9:** Basic parameters of fatigue-healing-fatigue test.

Asphalt Type	Fatigue Test Temperature/°C	Loading Frequency/Hz	Applied Strain/%	Damage Degree	Healing Temperature/°C	Healing Time/min
70#	25	10	4	50%	25, 35, 45	10,20,30,40,50,70
SBS, RAP					25, 45, 65	

**Table 10:** Basic parameters of fracture-healing-fracture test.

Asphalt Type	Loading Rate/mm·min^−1^	Healing Temperature/°C	Healing Time/h
70#	100	25, 35, 45	4,8,12
SBS, RAP		25, 45, 65	

## Data Availability

Data are contained within the article.
